# High self-efficacy – a predictor of reduced pain and higher levels of physical activity among patients with osteoarthritis: an observational study

**DOI:** 10.1186/s12891-020-03407-x

**Published:** 2020-06-13

**Authors:** Åsa Degerstedt, Hassan Alinaghizadeh, Carina A. Thorstensson, Christina B. Olsson

**Affiliations:** 1 Team Aktiv Primärvårdsrehab, Stockholm, Hässelby Sweden; 2 Academic Primary Healthcare Centre, Stockholm, Region Stockholm Sweden; 3 Institute of Neuroscience and Physiology, University of Gothenburg, Gothenburg, Sweden; 4 Department of Medicine, Karolinska Institutet, Stockholm, Solna Sweden; 5 Department of Neurobiology, Care Sciences and Society, Division of Physiotherapy, Karolinska Institutet, Stockholm, Sweden; 6 Danderyds akademiska vårdcentral, Golfvägen 8, 182 31, Danderyd, Sweden

**Keywords:** Exercise, Hip, Knee, Education, Primary health care

## Abstract

**Background:**

Self-efficacy is considered a core component in self-management. However, there is a lack of knowledge about the association between self-efficacy and health-related outcomes in osteoarthritis. The aim of this study was to investigate whether self-efficacy at baseline was associated with change over time in pain and physical activity after a supported osteoarthritis self-management programme.

**Methods:**

A total of 3266 patients with hip or knee osteoarthritis attended this observational, register-based study. Self-efficacy was assessed using the Arthritis Self-Efficacy Scale. Pain was estimated on a visual analogue scale and physical activity by self-reporting number of days per week the patients were physically active ≥30 min. Data were self-reported at baseline and at follow-ups after 3 and 12 months. Analyses were performed using a mixed linear model analysis and are presented with an unadjusted and an adjusted model.

**Results:**

High vs low self-efficacy for pain management at baseline resulted in reduced pain and increased physical activity at the follow-ups; least squares means and standard error were 37.43 ± 0.40 vs 44.26 ± 0.40, for pain, and 5.05 ± 0.07 vs 4.90 ± 0.08 for physical activity. High self-efficacy for management of other symptoms resulted in lower pain and higher physical activity at follow-up: 35.78 ± 0.71 vs 41.76 ± 0.71 for pain, and 5.08 ± 0.05 vs 4.72 ± 0.05 for physical activity. Patients with obesity reported lower activity levels at the follow-ups.

**Conclusion:**

Self-efficacy at baseline was associated with change over time in pain and physical activity at 3 and 12 months after the supported osteoarthritis self-management programme. High self-efficacy had a positive effect on pain and physical activity, indicating the need for exploring and strengthening patients’ self-efficacy. Patients with obesity may need further interventions and support during a self-management programme to achieve an increase in physical activity.

## Background

Osteoarthritis is one of the most common joint disorders worldwide [1], causing pain and stiffness, which can lead to inactivity, poor health and premature death [2, 3]. Basic treatment of osteoarthritis involves regular and lifelong physical activity and it is therefore essential that those affected can independently manage sustainable self-care including an active lifestyle.

Non-pharmacological guidelines for osteoarthritis include self-management and education, exercise, weight loss if overweight, and joint replacement where appropriate [4, 5]. Patient education, in the form of a supported self-management programme, designed to meet these guidelines, has been developed and implemented nationwide in Sweden [6]. The programme is based on theories of behavioural change and aims to provide patients with a sense of self-control and knowledge to adopt a healthy and active lifestyle.

Physical activity has a positive impact on physical function and disease-related symptoms such as pain [7–10]. Furthermore, physical activity is a strong, evidence-based measure for primary and secondary prevention of osteoarthritis, above all in the knee [11–13]. Still, many patients do not comply with exercise recommendations in the long term although they know about and have experienced the positive effects of physical activity and training [14–16]. The understanding of factors influencing physical activity behaviour is important for the development and improvement of health care interventions. Potential factors influencing adherence to physical activity are e.g. personal experiences, beliefs, attitudes and emotions, as well as the social environment, including healthcare and social support [17]. In addition, factors such as age, sex, health status, overweight, education level, and ethnic origin are associated with level of physical activity, together with self-efficacy, which is one of the clearest correlates of physical activity level in the general population [18, 19]. Self-efficacy has been defined as a person’s belief that they have the ability to accomplish or perform a task to achieve a desired outcome [20] such as reducing pain, or adapting daily activities to remain physically active despite pain and stiffness.

Self-efficacy is considered a core component in self-management [20, 21], yet there is a lack of knowledge about the association between self-efficacy and health-related outcomes in osteoarthritis. To our knowledge, previous longitudinal studies investigating the impact of factors such as self-efficacy on outcome are limited to knee osteoarthritis, include small populations or have an experimental design to test a hypothesis [22–24]. Hence, the aim of this study was to: (1) elucidate whether self-efficacy at baseline, before attending a supported osteoarthritis self-management programme, is associated with change over time in pain and physical activity after the programme; and (2) explore the impact of background factors such as age, gender, marital status, ethnicity, education level, body mass index (BMI), joint(s) affected by osteoarthritis, disease duration, and difficulty walking on patient-reported pain intensity and physical activity level.

## Methods

### Study design and procedures

**Fig. 1:**
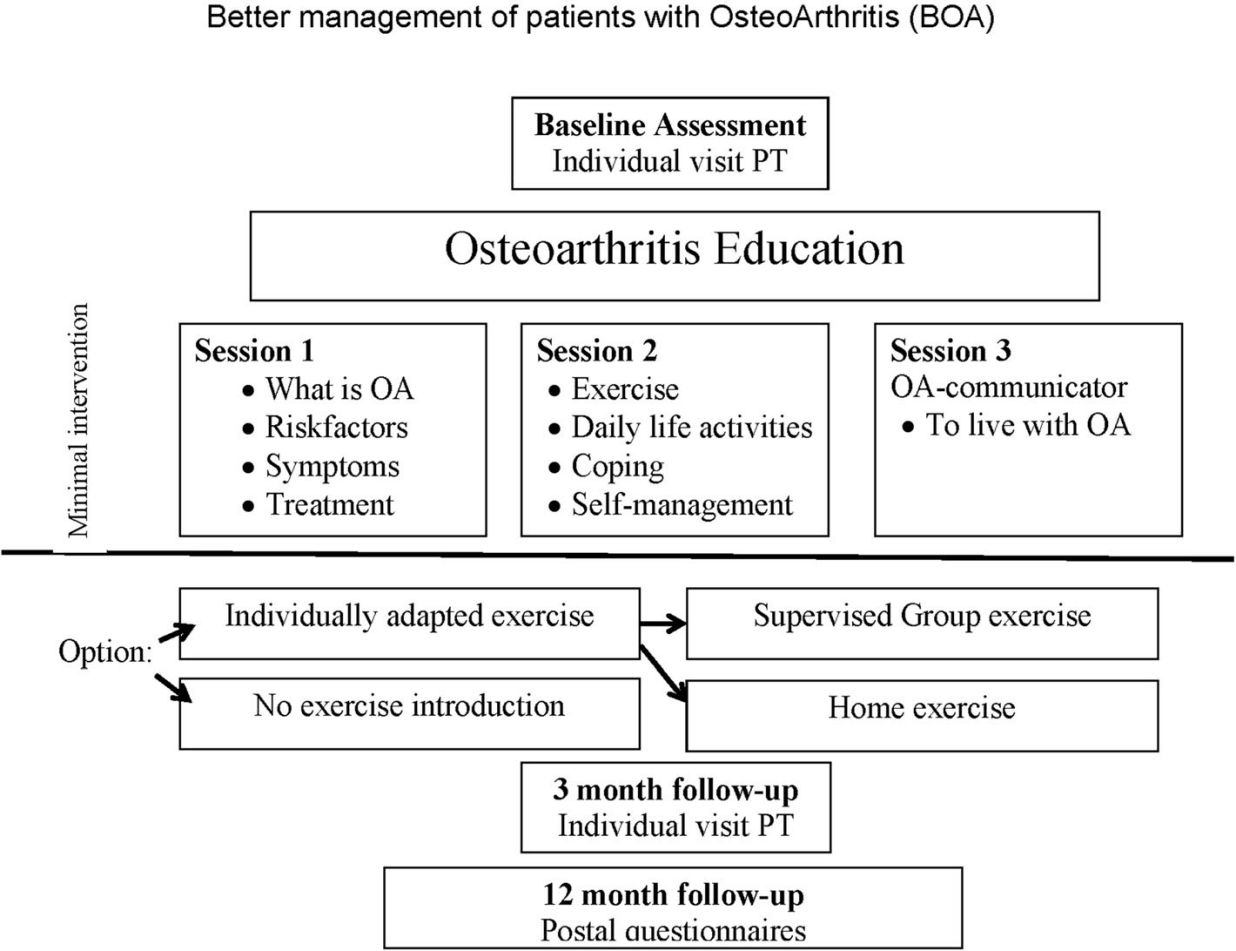
Disposition of the supported osteoarthritis self-management program for patients. OA = osteoarthritis; PT = physiotherapist

This is a prospective observational study using data from the National Quality Register for Better Management of Patients with Osteoarthritis (BOA), a register comprising patient-reported outcomes collected before and after a supported osteoarthritis self-management programme in primary care [6]. The programme is described in detail elsewhere [6]. In brief, it includes a minimum of two theoretical sessions of about 90 min each, in groups of between seven and 12 participants. The sessions, based on interactive discussions, are led by a physiotherapist in primary care and comprise information about osteoarthritis, available treatments, coping strategies, exercise, and self-management. All patients are offered an individually adapted exercise programme after completion of the theoretical sessions. Then they can opt to exercise on their own or under supervision of a physiotherapist together with others from the programme for 6 weeks (maximum twice a week), which means getting support, advice and individual adjustments to their programme. An individual follow-up is scheduled 3 months after the first visit, regardless of whether the patient chose to participate in exercise or not. (Fig. 1).

The present study includes data from patients who completed the 12-month follow-up between January 2008 and August 2012 and answered at least on one of the two outcomes, pain and physical activity. Both men and women were enrolled, of all ages, with unilateral or bilateral problems from the hip and/or knee. The diagnosis of osteoarthritis was verified by a physiotherapist after performing a clinical examination based on national guidelines [25]. Patients with inflammatory joint disease, malignancy and/or sequelae hip fracture and patients who do not read and write Swedish are not included in the register.

### Measurements

More than 400 primary care units in Sweden reported the data included in the BOA register. Patient-reported questionnaires were used at baseline and again 3 months later, after completion of the programme. After 12 months, the questionnaire was sent to the patients by mail. Descriptive data regarding date of birth, gender, marital status, ethnicity, education level, height and weight, joint(s) affected by osteoarthritis, disease duration, and difficulty walking due to osteoarthritis or other problems were collected at baseline by a physiotherapist (Table 1). Body mass index was calculated and categorized into: underweight < 18.5 kg/m2, normal weight 18.5–24.9 kg/m2, overweight 25–29.9 kg/m2, and obese ≥30 kg/m2 [26].

**Table 1:** Demographic and clinical characteristics at baseline (*n* = 3266)

Variable	Total	Low SE pain ≤62	High SE pain > 62	***p***-value	Low SE OS ≤68	High SE OS > 68	***p***-value
**Pain**^**a**^**, mean (SD)**							
baseline	48.48 (18.48)	52.96 (16.86)	43.46 (18.88)	< 0.01^c^*	52.60 (17.46)	43.86 (18.48)	< 0.01^c^*
3 months	37.68 (19.68)	42.35 (19.11)	32.56 (19.00)	< 0.01^c^*	42.17 (19.06)	32.92 (19.28)	< 0.01^c^*
1 year	39.97 (20.34)	44.24 (19.26)	35.23 (20.45)	< 0.01^c^*	43.96 (19.73)	35.54 (20.19)	< 0.01^c^*
**Physical activity**^**b**^**, mean (SD)**							
baseline	5.22 (2.05)	5.12 (2.15)	5.33 (1.92)	< 0.01^c^*	4.99 (2.17)	5.47 (1.87)	< 0.01^c^*
3 months	5.45 (1.76)	5.40 (1.81)	5.50 (1.70)	0.11^c^	5.29 (1.84)	5.60 (1.65)	< 0.01^c^*
1 year	5.14 (1.91)	5.06 (1.99)	5.24 (1.81)	< 0.01*^c^	4.99 (2.01)	5.31 (1.78)	< 0.01^c^*
**Age, yrs**							
mean (SD)	64.73 (9.22)	66.33 (9.30)	64.01 (9.08)	< 0.01*^c^	65.42 (9.57)	63.90 (8.74)	< 0.01^c^*
min**–**max	27–93	32–93	27–89		32–93	27–88	
**Gender,*n*(%)**							
male	923 (28.26)	495 (28.98)	420 (27.50)	0.35^d^	465 (27.26)	442 (29.33)	0.19^d^
female	2343 (71.74)	1213 (71.5)	1107 (72.50)		1241 (72.74)	1065 (70.67)	
**Marital status,*n*(%)**							
cohabiting	2419 (74.29)	1235 (72.48)	1161 (76.23)	0.01^d^*	1208 (70.98)	1168 (77.66)	< 0.01^d^*
living alone	837 (25.71)	469 (27.52)	362 (23.77)		494 (29.02)	336 (22.34)	
**Born in Sweden,*n*(%)**							
yes	3013 (92.37)	1569 (91.92)	1419 (92.99)	0.25^d^	1559 (91.44)	1410 (93.63)	0.02^d^*
no	249 (7.63)	138 (8.08)	107 (7.01)		146 (8.56)	96 (6.37)	
**Education,*n*(%)**							
compulsory school	1213 (37.37)	714 (42.02)	484 (31.86)	< 0.01^d^*	710 (41.89)	478 (31.85)	< 0.01^d^*
upper secondary school	1118 (34.44)	601 (35.37)	514 (33.84)		594 (35.04)	512 (34.11)	
university	915 (28.19)	384 (22.60)	521 (34.30)		391 (23.07)	511 (34.04)	
**BMI, kg/m**^**2**^**,*n*(%)**							
underweight < 18.5	64 (2.01)	34 (2.04)	27 (1.82)	0.04^d^*	33 (1.99)	27 (1.84)	< 0.01^d^*
normal weight 18.5**–**24.9	877 (27.59)	425 (25.56)	442 (29.76)		426 (25.72)	436 (29.66)	
overweight 25**–**29.9	1338 (42.09)	706 (42.45)	618 (41.62)		678 (40.94)	636 (43.27)	
obese ≥30	900 (28.31)	498 (29.95)	398 (26.80)		519 (31.34)	371 (25.24)	
**Most affected joint,*n*(%)**							
hip	894 (27.37)	498 (29.16)	388 (25.41)	0.02^d^*	492 (28.84)	388 (25.75)	0.05^d^
knee	2372 (72.63)	1210 (70.84)	1139 (74.54)		1214 (71.16)	1119 (74.25)	
**Affected other hip/knee,*n*(%)**							
no	1459 (45.13)	701 (41.55)	746 (49.18)	< 0.01^d^*	727 (43.12)	713 (47.63)	0.01^d^*
yes	1774 (54.87)	986 (58.45)	771 (50.82)		959 (56.88)	784 (52.37)	
**Walking difficulty,*n*(%)**							
no	539 (16.70)	176 (10.44)	357 (23.58)	< 0.01^d^*	178 (10.58)	352 (23.53)	< 0.01^d^*
yes	2689 (83.3)	1510 (88.1)	1157 (76.42)		1505 (89.42)	1144 (76.47)	

Demographic and clinical characteristics at baseline for the total cohort (*n* = 3266) and separately for the groups of participants with low vs high self-efficacy for pain management and low vs high self-efficacy for management of other symptoms

*SD* standard deviation, *SE pain* self-efficacy for pain management, *SE OS* self-efficacy for management of other symptoms, *VAS* visual analogue scale

^a^Measured using a VAS from 0 to 100; ^b^days per week of being physically active for ≥30 min; ^c^*t*-test; ^d^ chi-square test

*p-value< 0.05

Self-efficacy was assessed using two subscales of the Swedish version of the Arthritis Self-Efficacy Scale (ASES-S) for pain and other symptoms related to arthritis [27, 28]. Each question was answered on a Likert scale from 10 (very uncertain) to 100 (very certain) and the patients were asked to circle the number that best described their confidence in their ability to manage symptoms of arthritis. The third sub scale of ASES-S, self-efficacy for activities of daily living, is not included in the BOA register.

Pain was measured by a visual analogue scale, from 0 (no pain) to 100 (worst possible pain) [29]. The patients were asked to rate the average pain from their most troublesome joint(s) during the last month.

Physical activity was defined for the patients as any activity that causes the heart to beat faster and makes you breathless and warm (e.g. walking, cycling, dancing, vacuuming or gardening). Self-reported physical activity was measured as number of days per week with 30 min or more of physical activity (0–7 days/week).

### Statistical analysis

The variables self-efficacy for pain management and self-efficacy for managing other symptoms were dichotomized into low and high self-efficacy by dividing the sum scores using medians as cut-off points. This resulted in two groups for self-efficacy for pain management: ≤62 (low pain management self-efficacy) and > 62 (high pain management self-efficacy), and two groups for self-efficacy for managing other symptoms: ≤68 (low symptom management self-efficacy) and > 68 (high symptom management self-efficacy). Group comparison of demographic and background data at baseline between those with low and high self-efficacy was conducted by t-test where normality assumptions were not violated. The categorical level of background factors was evaluated by Chi-Square test.

A mixed linear model analysis with restricted maximum likelihood method was used to evaluate the association between self-efficacy for pain management and for management of other symptoms at baseline, and pain and physical activity levels at the follow-ups. A mixed model analysis of longitudinal data was chosen since it allowed us to include patients with missing data [30]. The correlation between self-efficacy for pain management and self-efficacy for managing other symptoms at baseline was *r* = 0.76 (*p* < 0.001). Due to multicollinearity, a separate model was conducted for each of the two independent variables in combination with the two outcomes (pain and physical activity), resulting in four models [31]. Each of the outcomes, pain and physical activity, was used computing for random intercept at individual level as level-1, and random slope of time at self-efficacy for managing pain and self-efficacy for managing other symptoms as level-2 [32, 33]. The respective mixed models were conducted with 10 confounding factors included as fixed effects. Confounding factors found to have statistical significance in the primary unadjusted models (gender, age, birth place, education, marital status, affected other hip/knee, duration, most affected joint hip/knee, walking difficulty, and BMI) were then tested in subsequent adjusted models for interaction with self-efficacy for pain management and self-efficacy for managing other symptoms. The results were simplified and presented for each outcome over time as estimated least squares means and standard error. To perform a suitable test for a higher-order effect among self-efficacy for pain management and self-efficacy for management of other symptoms across groups over time, the Least Squares Means was grouped into subsets of time, which is known as an analysis of simple effect [34]. Differences among time periods between groups were tested by contrast test. Interaction between exposure and time was controlled in all adjusted models (data not shown).

Effect size correlation and standardized units of difference (f^2^) were used to explain the magnitude of the confounders and the association with outcome at the individual level over time, taking into account self-efficacy for management of pain and other symptoms at baseline. The observed effect size can provide valuable information to help evaluate the magnitude of important confounders over time adjusting for the two exposures self-efficacy for pain management and self-efficacy for management of other symptoms. According to Cohen, the effect size of 0.02–0.15 indicates a small effect, while 0.15–0.35 indicates a medium and > 0.35 a large effect [35]. The proportion of variance (R^2^: Estimated Covariance Parameter) explained by the full respectively empty model in each step was derived using the mixed model approach and was used to calculate effect size of each potential confounder across all measurement times [36].

$$ {f}^2=\frac{R^2}{1-{R}^2} $$

All analyses were performed using SAS 9.4 and a *p*-value of 0.05 was used to reject the null hypothesis [30].

## Results

**Fig. 2:**
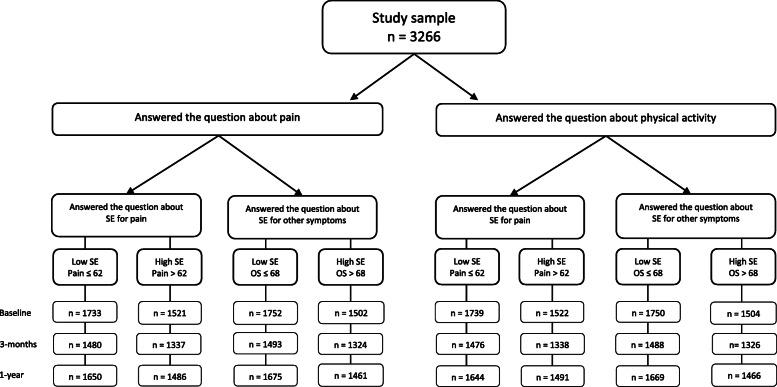
Flow chart of the study, showing the number of participants that answered at baseline and at follow-up. SE = self-efficacy; SE pain = self-efficacy for pain management; SE OS = self-efficacy for management of other symptoms

A total of 3266 patients were included in this study, 352 of whom did not respond to the 3- month follow-up. (Fig. 2). There were no significant differences in background variables between patients who answered (*n* = 2914) and patients who did not answer the follow-up questions (*n* = 352) except for the questions regarding walking difficulties (*p* = 0.048) and BMI (*p* < 0.01). Those who did not answer had more walking difficulties (9% vs 12%). For BMI, there were more patients with underweight (4.1% vs 1.8%) and obesity (31.1% vs 28%) but fewer patients with overweight (37% vs 42.7%) among those who did not respond. The mean age of the study population was 64.7 (range 27–93) years and 71.7% were women.

Characteristics of the whole population as well as of the separate groups with low respectively high self-efficacy are shown in Table 1.

Self-efficacy for pain management at baseline was significantly associated with change over time in pain and physical activity. The adjusted models show that patients with high self-efficacy, compared with patients with low self-efficacy, reported lower pain intensity (least squares means±standard error 37.43 ± 0.40 and 44.26 ± 0.40, respectively; *p* < 0.01) and higher physical activity at the follow-ups (least squares means±standard error 5.05 ± 0.07 and 4.90 ± 0.08, respectively; *p* < 0.01) (Table 2).

**Table 2:** Self-efficacy for pain at baseline, association with pain and physical activity at the follow-ups

	Pain^**a**^					Physical activity^**b**^				
	Unadjusted model^c^			Adjusted model^c^		Unadjusted model^c^			Adjusted model^c^	
	LSM ± SE	***p***-value	Effect size (***f***^***2***^)^d^	LSM ± SE	***p***-value	LSM ± SE	***p***-value	Effect size (***f***^***2***^)^d^	LSM ± SE	***p***-value
**Intercept**				31.67 ± 0.81	< 0.01*				5.27 ± 0.15	< 0.01*
**Time**					< 0.01*					< 0.01*
**1 = Baseline (ref)**				47.98 ± 0.40					4.92 ± 0.08	
**2 = 3 months**				37.07 ± 0.41					5.15 ± 0.08	
**3 = 1 year**				37.47 ± 0.43					4.86 ± 0.08	
**Self-efficacy for pain management**					< 0.01*					< 0.01*
**0 = ≤62 (ref)**				44.26 ± 0.40					4.90 ± 0.08	
**1 = > 62**				37.43 ± 0.40					5.05 ± 0.07	
**Gender**		< 0.01*	0.002		< 0.01*		< 0.01	0.010		< 0.01*
**1 = Male**	42.81 ± 0.28			40.25 ± 0.42		4.98 ± 0.03			4.73 ± 0.05	
**2 = Female (ref)**	43.99 ± 0.18			41.45 ± 0.38		5.37 ± 0.05			5.07 ± 0.04	
**Age, yrs**		0.05	0.001		0.06		< 0.01	0.003		< 0.01*
**1 = ≤45 (ref)**	44.80 ± 0.81			41.21 ± 0.72		4.63 ± 0.16			4.54 ± 0.08	
**2 = 46–75**	43.77 ± 0.16			41.09 ± 0.32		5.27 ± 0.03			5.06 ± 0.04	
**3 = > 75**	42.95 ± 0.39			40.26 ± 0.43		5.32 ± 0.08			5.10 ± 0.05	
**Born in Sweden**		< 0.01*	0.002		< 0.01*		0.08	0.001		0.10
**0 = No**	48.26 ± 0.53			42.61 ± 0.52		5.10 ± 0.10			4.79 ± 0.06	
**1 = Yes (ref)**	43.29 ± 0.15			39.09 ± 0.33		5.27 ± 0.03			5.01 ± 0.04	
**Marital status**		< 0.01*	0.001		0.13		0.04	0.001		0.08
**1 = Cohabiting (ref)**	43.35 ± 0.17			40.65 ± 0.39		5.30 ± 0.03			4.90 ± 0.05	
**2 = Living alone**	44.47 ± 0.28			41.05 ± 0.42		5.36 ± 0.05			4.89 ± 0.05	
**Education**		< 0.01*	0.001		< 0.01*		0.09	0.000		0.15
**1 = Compulsory school (ref)**	44.41 ± 0.24			41.81 ± 0.42		5.32 ± 0.04			4.92 ± 0.05	
**2 = Upper secondary school**	44.59 ± 0.25			42.06 ± 0.40		5.18 ± 0.05			4.88 ± 0.05	
**3 = University**	41.39 ± 0.28			38.68 ± 0.42		5.27 ± 0.05			4.89 ± 0.05	
**Affected other hip/knee**		< 0.01*	0.001		< 0.01*		0.81	0.000		0.79
**0 = No**	41.36 ± 0.22			39.00 ± 0.40		5.27 ± 0.04			4.89 ± 0.05	
**1 = Yes (ref)**	45.86 ± 0.21			42.70 ± 0.40		5.25 ± 0.04			4.90 ± 0.05	
**Most affected joint**		< 0.01*	0.001		< 0.01*		0.08	0.001		0.61
**1 = Hip (ref)**	45.36 ± 0.27			42.68 ± 0.42		5.32 ± 0.04			4.89 ± 0.05	
**2 = Knee**	42.94 ± 0.18			39.02 ± 0.39		5.25 ± 0.02			4.90 ± 0.05	
**Difficulty walking**		< 0.01*	0.025		< 0.01*		< 0.01	0.002		0.03*
**0 = No (ref)**	32.21 ± 0.36			34.86 ± 0.45		5.46 ± 0.05			4.84 ± 0.04	
**1 = Yes**	45.81 ± 0.16			46.82 ± 0.37		5.23 ± 0.02			4.95 ± 0.05	
**BMI, kg/m**^**2**^		< 0.01*	0.008		0.05		< 0.01	0.054		< 0.01*
**1 = Underweight < 18.5**	43.21 ± 0.92			40.40 ± 0.82		5.31 ± 0.14			4.74 ± 0.10	
**2 = Normal weight 18.5–24.9 (ref)**	40.61 ± 0.29			38.83 ± 0.38		5.57 ± 0.04			5.28 ± 0.05	
**3 = Overweight 25–29.9**	43.06 ± 0.22			40.45 ± 0.36		5.33 ± 0.03			5.00 ± 0.04	
**4 = Obese ≥ 30**	47.40 ± 0.28			43.70 ± 0.39		4.86 ± 0.04			4.56 ± 0.05	

Self-efficacy for pain management at baseline, and association with pain and physical activity at the follow-ups. Estimated least squares means from unadjusted and adjusted models for pain and physical activity using self-efficacy for pain management as a fixed factor in all models. Effect sizes are from the unadjusted model for each demographic and clinical characteristic

*BMI* body mass index, *LSM* least squares means, *ref.* reference category, *SE* standard error

^a^Measured using a VAS from 0 to 100; ^b^days per week of being physically active for ≥30 min; ^c^Mixed linear model analysis with a restricted maximum likelihood method; ^d^effect size interpreted according to Cohen’s guidelines: ≥0.02 = small; ≥0.15 = medium; ≥0.35 = large [35]

*p-value< 0.05

**Fig. 3:**
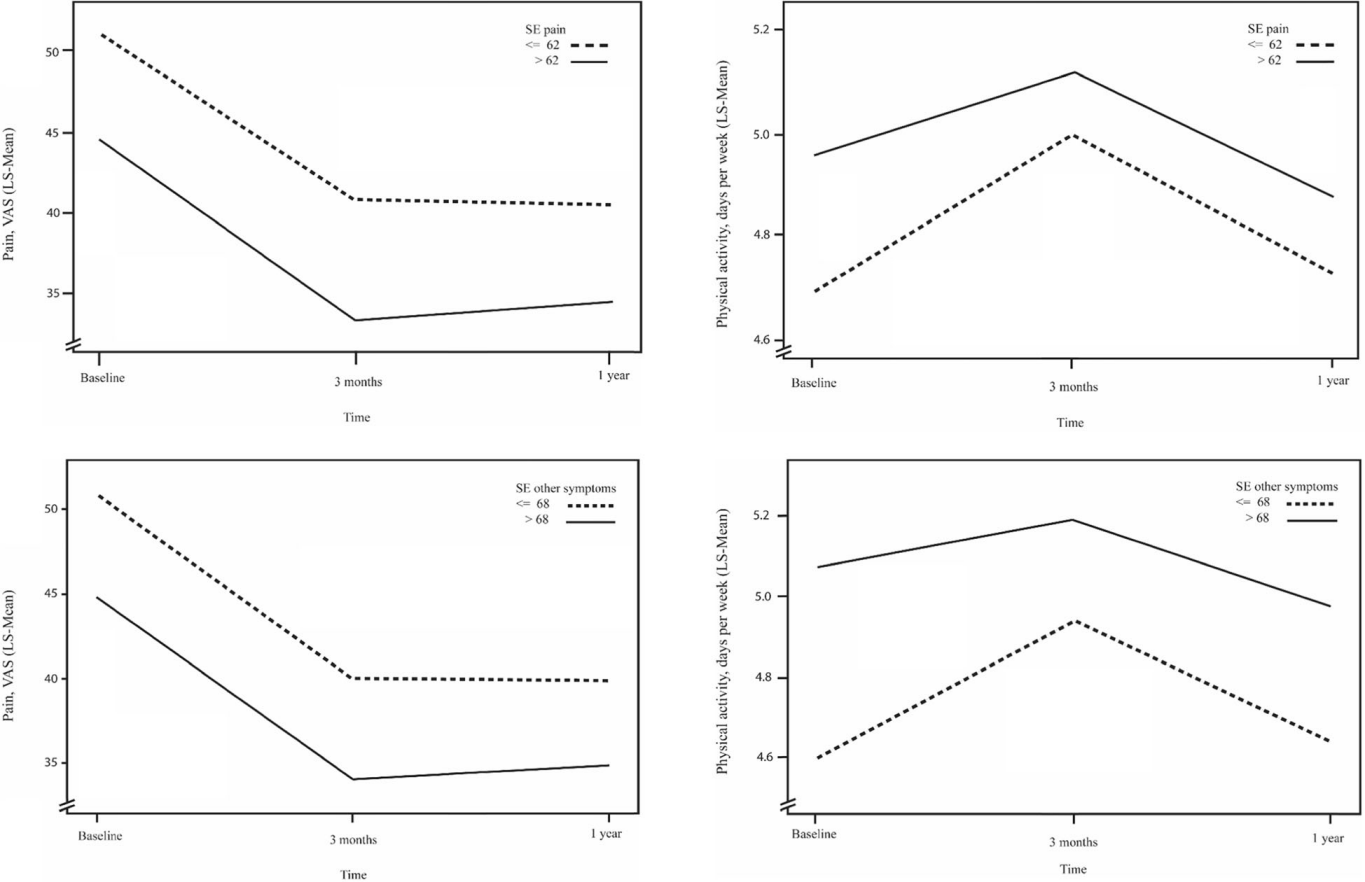
Least Squares Means (LS-mean) adjusted for all confounders in mixed linear models for Pain (VAS scale) and Physical activity (days per week being physically active for at least 30 min) at baseline and follow-ups for patients with high and low self-efficacy for pain (SE pain) and other symptoms (SE other symptoms) at baseline

Self-efficacy for management of other symptoms at baseline was significantly associated with change over time in pain and physical activity. The adjusted models show that patients with high self-efficacy for management of other symptoms reported lower pain intensity at follow-up than did patients with low self-efficacy (least squares means±standard error 35.78 ± 0.71 and 41.76 ± 0.71, respectively; *p* < 0.01) and that they also reported higher physical activity levels (least squares means±standard error 5.08 ± 0.05 and 4.72 ± 0.05, respectively; *p* < 0.01) (Table 3). Change over time for pain and physical activity with three measurement points are shown in Fig. 3.

**Table 3:** Self-efficacy for other symptoms at baseline, association with pain and physical activity at the follow-ups

	Pain^**a**^					Physical activity^**b**^				
	Unadjusted model^c^			Adjusted model^c^		Unadjusted model^c^			Adjusted model^c^	
	LSM ± SE	p-value	Effect size (***f***^***2***^)^b^	LSM ± SE	***p***-value	LSM ± SE	p-value	Effect size (***f***^***2***^)^b^	LSM ± SE	p-value
**Intercept**				27.91 ± 1.39	< 0.01*				5.39 ± 0.09	< 0.01*
**Time**					< 0.01*					< 0.01*
**1 = Baseline (ref)**				45.14 ± 0.73					4.84 ± 0.05	
**2 = 3 months**				34.51 ± 0.74					5.07 ± 0.05	
**3 = 1 year**				36.67 ± 0.73					4.80 ± 0.05	
**Self-efficacy for management of other symptoms**					< 0.01*					< 0.01*
**0 = ≤68 (ref)**				41.76 ± 0.71					4.72 ± 0.05	
**1 = > 68**				35.78 ± 0.71					5.08 ± 0.05	
**Gender**		< 0.01*	0.001		< 0.01*		< 0.01*	0.003		< 0.01*
**1 = Male**	40.43 ± 0.51			37.63 ± 0.75		4.98 ± 0.03			4.73 ± 0.05	
**2 = Female (ref)**	42.86 ± 0.32			39.92 ± 0.69		5.32 ± 0.02			5.08 ± 0.04	
**Age, yrs**		0.07	0.001		< 0.01*		< 0.01*	0.002		< 0.01*
**1 = ≤45 (ref)**	39.13 ± 1.62			37.27 ± 1.28		4.68 ± 0.09			4.54 ± 0.08	
**2 = 46–75**	42.39 ± 0.29			40.14 ± 0.58		5.37 ± 0.02			5.12 ± 0.05	
**3 = > 75**	41.31 ± 0.79			38.92 ± 0.79		5.37 ± 0.05			5.11 ± 0.05	
**Born in Sweden**		< 0.01*	0.002		< 0.01*		< 0.01*	0.001		< 0.01*
**0 = No**	47.96 ± 0.98			41.13 ± 0.92		4.98 ± 0.06			4.80 ± 0.06	
**1 = Yes (ref)**	41.71 ± 0.28			36.41 ± 0.61		5.25 ± 0.02			5.01 ± 0.04	
**Marital status**		0.22	0.001		0.96		0.32	0.001		0.97
**1 = Cohabiting (ref)**	41.99 ± 0.31			38.78 ± 0.70		5.21 ± 0.02			4.90 ± 0.05	
**2 = Living alone**	42.75 ± 0.54			38.76 ± 0.74		5.25 ± 0.03			4.90 ± 0.05	
**Education**		< 0.01*	0.001		< 0.01*		0.01*	0.000		0.10
**1 = Compulsory school (ref)**	42.92 ± 0.44			39.70 ± 0.74		5.26 ± 0.03			4.94 ± 0.05	
**2 = Upper secondary school**	43.29 ± 0.46			40.17 ± 0.72		5.16 ± 0.03			4.88 ± 0.05	
**3 = University**	39.72 ± 0.51			36.45 ± 0.76		5.26 ± 0.03			4.88 ± 0.05	
**Affected other hip/knee**		< 0.01*	0.002		< 0.01*		0.47	−0.000		0.64
**0 = No**	39.39 ± 0.40			36.89 ± 0.72		5.23 ± 0.02			4.90 ± 0.05	
**1 = Yes (ref)**	44.42 ± 0.36			40.66 ± 0.71		5.21 ± 0.02			4.91 ± 0.05	
**Most affected joint**		< 0.01*	0.000		< 0.01*		0.15	0.000		0.80
**1 = Hip (ref)**	43.51 ± 0.52			40.42 ± 0.75		5.26 ± 0.03			4.90 ± 0.05	
**2 = Knee**	41.67 ± 0.32			37.13 ± 0.69		5.21 ± 0.02			4.91 ± 0.05	
**Walking difficulty**			0.025		< 0.01*		< 0.01*	0.001		0.08
**0 = No (ref)**	31.08 ± 0.64	< 0.01*		32.87 ± 0.80		5.36 ± 0.04			4.94 ± 0.05	
**1 = Yes**	44.41 ± 0.28			44.67 ± 0.67		5.20 ± 0.02			4.87 ± 0.04	
**BMI, kg/m**^**2**^		< 0.01*	0.007		< 0.01*		< 0.01*	0.052		< 0.01*
**1 = Underweight < 18.5**	41.29 ± 1.92			37.60 ± 1.59		5.08 ± 0.10			4.76 ± 0.10	
**2 = Normal weight 18.5–24.9 (ref)**	39.41 ± 0.51			37.30 ± 0.63		5.59 ± 0.03			5.27 ± 0.04	
**3 = Overweight 25–29.9**	41.26 ± 0.42			38.18 ± 0.63		5.26 ± 0.03			5.00 ± 0.04	
**4 = Obese ≥ 30**	46.59 ± 0.51			42.01 ± 0.67		4.84 ± 0.03			4.58 ± 0.05	

Self-efficacy for management of other symptoms at baseline, and association with pain and physical activity at the follow-ups. Estimated least squares means from unadjusted and adjusted models for pain and physical activity using self-efficacy for management of other symptoms as a fixed factor in all models. Effect sizes are from the unadjusted model for each demographic and clinical characteristic

*BMI* body mass index, *LSM* least squares means, *ref.* reference category, *SE* standard error

^a^Measured using a VAS from 0 to 100; ^b^days per week of being physically active for ≥30 min; ^c^Mixed linear model analysis with a restricted maximum likelihood method; ^d^effect size interpreted according to Cohen’s guidelines: ≥0.02 = small; ≥0.15 = medium; ≥0.35 = large [35]

*p-value< 0.05

Body mass index in association with self-efficacy for pain management as well as for other symptom management was the strongest confounding factor for physical activity level (effect size 0.054 and 0.052, respectively). Patients with normal body weight were more physically active in comparison with underweight, overweight and obese patients (Tables 2 and 3).

## Discussion

The main aim of this study was to investigate whether self-efficacy measured at baseline, adjusting for potential confounders, was associated with change over time in pain and physical activity among patients with hip and/or knee osteoarthritis after attending a supported self-management programme.

In the present study, self-efficacy for managing other symptoms had a stronger association with change over time in pain intensity, compared with self-efficacy for pain management. It could be that some symptoms are less easy to influence, and it is possible that those reporting high self-efficacy for managing other symptoms at baseline did not experience symptoms such as fatigue, depression and frustration.

The levels of physical activity for the whole population had increased by the first follow-up but then dropped again after 12 months to more or less the same level as at baseline. In a study by Baruth and Wilcox [37], people with suboptimal self-efficacy at baseline who achieved an increase in self-efficacy during the reported intervention had a better chance to reach recommended levels of physical activity. One study found that self-efficacy for management of both pain and other symptoms increased during the osteoarthritis self-management programme but returned to the baseline level by the 12-month follow-up. Age, intervention type, osteoarthritis location and BMI seemed to influence the change in self-efficacy [38]. In the supported osteoarthritis self-management programme reported here the patients receive information about the disease and are supported in self-management through physical activity [14]. A trained physiotherapist supervising exercises and providing encouragement as well as answering questions and helping with individual adjustments can help to enhance self-efficacy [39, 40]. Patients have different motives for exercise behavior and thus different needs for support to enhance their self-efficacy [41]. There are several strategies that can be used in the clinic to help enhancing patients’ self-efficacy. Not only is it important to actively listen to and support the patient in developing a personalized plan to address potential barriers but also they should be encouraged to use self-management strategies [42]. Personal experience also helps build self-efficacy, as well as watching similar others engage in pain self-management strategies and successful behaviours [20, 40, 42].

Variables predicting a durable outcome may vary depending on the patient’s level of self-efficacy at baseline [37]. Osteoarthritis severity directly influences self-efficacy [43] and self-efficacy scores in osteoarthritis patients vary greatly according to the status of the studied population [38, 44]. Patients with low education, and/or those with difficulties walking, comorbidity that affects walking ability, and/or low physical activity reported lower self-efficacy at baseline, before entering the supported osteoarthritis self-management programme [38]. An increased focus on these patients to enhance their self-efficacy may result in better outcomes of the intervention. In building one’s self-efficacy it is encouraging to watch others with similar or slightly higher skills adopt successful behaviours and self-management strategies [40].

There are many variables that might be related to physical activity. For example, increasing age and female gender have been negatively associated with participating in physical activity for people with knee osteoarthritis in cross-sectional studies [45]. In the present study the effect of the included confounders on physical activity was small, indicating that other factors might be of importance for determining physical activity level in people with osteoarthritis. A systematic review of qualitative studies [17] found a complex interaction of physical as well as personal factors, including psychological, social and environmental factors that facilitate or hinder physical activity engagement in people with osteoarthritis.

Body mass index was the most important confounder in the unadjusted physical activity models; physical activity was lower at follow-up in the group with BMI ≥30 kg/m2. It has been shown that self-efficacy in obese patients with osteoarthritis decreases more over time than in those with lower BMI [38]. The reason might be exercise-related pain, co-morbidity or lack of inner motivation, but nonetheless it may signal a need to target individuals with high BMI and to find specific intervention strategies to promote self-efficacy and maintenance of long-term physical activity for this group [46].

Some strengths and limitations should be mentioned. This observational study is based on a large number of participants of different age, sex and ethnicity, from clinical practice in primary care all over Sweden. This increases the generalizability of the results and creates opportunities for a study of high power. In the present study the local or therapist-related variations of the intervention cannot be determined. Physiotherapists supervising exercises might be a confounder, affecting patients both positively and negatively. These kinds of personal factors were not adjusted for in this study. Both walking difficulties and underweight can be related to comorbidity, which may have influenced the lower participation at the individual follow-up after 3 months.

There is a risk that the question about how many days the patients were physically active may have been too vague to show accurate results. Overestimation has been found to result when self-reporting physical activity [47] and use of an objective instrument for registration of physical activity would have strengthened the reliability and validity of the measurements. However, this was not suitable for this kind of study where hundreds of clinics reported data to the BOA registry. A construct such as self-efficacy can only be collected using self-reporting of any kind, since it is a matter of a self-perceived ability. The complexity of the concept of self-efficacy has been debated and there has been some discussion whether the instrument Arthritis Self-Efficacy Scale really does measure self-efficacy [48, 49]. Furthermore, a review [50] found methodological weaknesses and poor evidence regarding the Arthritis Self-Efficacy Scale. However, it has been suggested that the scale is appropriate for use in research because of good reliability, validity, and change with interventions [50]. The Arthritis Self-Efficacy Scale score might vary naturally in the studied population [44] and there are no established cut-off points. In the present study medians were used to dichotomize into low and high self-efficacy, which might have affected the generalizability negatively.

The number of individuals differed between times for different variables. By using Mixed linear model for analyses all observations were kept in the model, compared to repeated measures like ANCOVA and MANCOVA, where observations with missing data are eliminated [30, 32, 33].

## Conclusions

Self-efficacy at baseline was associated with change over time in pain and physical activity at follow-up after the intervention. High self-efficacy had a positive effect on pain and physical activity, indicating the need for exploring and strengthening patients’ self-efficacy. Self-efficacy in combination with body mass index seems to be of importance; patients with obesity reported lower activity levels at follow-up, indicating that this group may need further interventions to achieve long-term results. These results need to be considered in clinical practice when developing interventions and treatment for osteoarthritis.

## Data Availability

The dataset generated and/or analyzed during the current study is governed by Region Västra Götaland. The authors are not allowed to share the data. The data is available from Center of Registers Västra Götaland, Gothenburg, Sweden (contact: boa@registercentrum.se) for researchers who meet the criteria for access to confidential data according to Swedish law.

## Abbreviations

BOA: Better management of patients with Osteoarthritis
BMI: Body Mass Index
